# Book Notices

**Published:** 1893-12

**Authors:** 


### Book Notices.

ESSENTIALS OF MINOR SURGERY, BANDAGING, AND VENEREAL DISEASES. Arranged in the form of questions and answers. Prepared especially for students of medicine. By Edward Martin, A. M., M. D., Clinical Professor of Genito-Urinary Diseases; Instructor in Operative Surgery, and Lecturer on Minor Surgery, University of Pennsylvania. Second edition, revised and enlarged; 166 pages; 78 illustrations. Price, cloth, $1. W. B. Saunders, Publisher, 925 Walnut street, Philadelphia.

Saunders' "Question Compends" have become very popular with medical students, and there is no denying the fact that they render him valuable assistance when preparing for the quiz or the "green room." Much valuable information is gained from them, and in a very practical and compact form. The one under consideration, No. 12, has been thoroughly revised and brought up to date. The latest appliances in the way of bandages, strappings, sutures, antiseptic dressings, etc., are given, and illustrations appear wherever necessary to elucidate the text. The book has its place, and is worth more than the price asked. H.

---

LEONARD'S PHYSICIAN'S POCKET DAY-BOOK. Bound in Red Morocco, with Flap, Pocket and Pencil Loop. Price, postpaid, $1. Published annually, by the Illustrated Medical Journal Co., Detroit, Mich.

This popular day-book is now in its 16th year of publication. It is good for *thirteen* months from the first of *any* month that it may be begun, and accommodates charges for fifty patients daily for that time, besides having cash department, and complete obstetric records. There is space for the diagnosis of each case, or for brief records of the treatment adopted, following each name-space. Name of each patient needs to be written but three times in a month. In has the usual *printed matter*, such as dose list, poisons and antidotes, urinary tests, exanthematicæ, disinfectants, weights and measures. The book is 7½ inches long and 3½ inches wide, so that it will carry bill-heads or currency bills without folding. It is bound in flexible covers, and weighs but five ounces, so that it is easily carried in the pocket.

